# SpeedyGenes: an improved gene synthesis method for the efficient production of error-corrected, synthetic protein libraries for directed evolution

**DOI:** 10.1093/protein/gzu029

**Published:** 2014-08-09

**Authors:** Andrew Currin, Neil Swainston, Philip J. Day, Douglas B. Kell

**Affiliations:** 1 Manchester Institute of Biotechnology, The University of Manchester, Manchester M1 7DN, UK; 2 School of Chemistry, The University of Manchester, Manchester M13 9PL, UK; 3 School of Computer Science, The University of Manchester, Manchester M13 9PL, UK; 4 Faculty of Medical and Human Sciences, The University of Manchester, Manchester M13 9PT, UK

**Keywords:** directed evolution, error correction, gene synthesis, protein libraries

## Abstract

The *de novo* synthesis of genes is becoming increasingly common in synthetic biology studies. However, the inherent error rate (introduced by errors incurred during oligonucleotide synthesis) limits its use in synthesising protein libraries to only short genes. Here we introduce SpeedyGenes, a PCR-based method for the synthesis of diverse protein libraries that includes an error-correction procedure, enabling the efficient synthesis of large genes for use directly in functional screening. First, we demonstrate an accurate gene synthesis method by synthesising and directly screening (without pre-selection) a 747 bp gene for green fluorescent protein (yielding 85% fluorescent colonies) and a larger 1518 bp gene (a monoamine oxidase, producing 76% colonies with full catalytic activity, a 4-fold improvement over previous methods). Secondly, we show that SpeedyGenes can accommodate multiple and combinatorial variant sequences while maintaining efficient enzymatic error correction, which is particularly crucial for larger genes. In its first application for directed evolution, we demonstrate the use of SpeedyGenes in the synthesis and screening of large libraries of MAO-N variants. Using this method, libraries are synthesised, transformed and screened within 3 days. Importantly, as each mutation we introduce is controlled by the oligonucleotide sequence, SpeedyGenes enables the synthesis of large, diverse, yet controlled variant sequences for the purposes of directed evolution.

## Introduction

The ability to synthesise *de novo* and to assemble DNA molecules of any desired sequence is a fundamental feature of synthetic biology and biotechnology (Tian *et al.*, 2009; Notka *et al.*, 2011; Ma *et al.*, 2012*a*). Applications of DNA synthesis and assembly are far reaching, including the engineering of proteins and cellular metabolism (Ellis *et al.*, 2011; Wang *et al.*, 2011), synthesis of genomes (Gibson *et al.*, 2008, 2009, 2010) and data storage (Goldman *et al.*, 2013). Consequently, the accurate and efficient synthesis of DNA molecules is a fundamental part of current biological research. To achieve this, sequences are designed *in silico* to facilitate efficient synthesis *in vitro* and these two (computational and experimental) facets should be both complementary and integrated.

Following the design and synthesis of oligonucleotides, full-length genes can be assembled using PCR-based (Stemmer *et al.*, 1995; Gao *et al.*, 2003; Xiong *et al.*, 2004; Cherry *et al.*, 2008) or ligation-based (Smith *et al.*, 2003; Bang and Church, 2008; Horspool *et al.*, 2010; Yang *et al.*, 2012) methods. Regardless of the synthesis method, incorrect bases (arising during the chemical synthesis of the oligonucleotides) are likely to be incorporated into the gene sequence during assembly. Consequently each copy of the synthesised gene often encodes randomly placed mutations or (more commonly) base insertions or deletions, with a greater number incorporated as the length of the synthesised nucleic acid increases. These errors require removal to realise the desired DNA sequence (Xiong *et al.*, 2008; Ma *et al.*, 2012*b*), a process that creates a significant bottleneck in efficient gene synthesis. Various strategies have been employed to reduce encoded errors, including use of mismatch-binding proteins (Carr and Church, 2009), enzymatic mismatch cleavage (Fuhrmann *et al.*, 2005; Saaem *et al.*, 2012), site-directed mutagenesis (Xiong *et al.*, 2008), synthesis of higher quality oligonucleotides (LeProust *et al.*, 2010) and retrieval of sequence-verified molecules using pyrosequencing (Matzas *et al.*, 2010). However, none of these methods is entirely satisfactory, nor (in particular) is easily applicable to the production of genetic libraries.

Enzymatic mismatch cleavage, which is the focus of this work, utilises the endonuclease function of mismatch-repair enzymes to cleave the DNA strand specifically at an error; the erroneous nucleotides are then removed by the proofreading activity of a high-fidelity polymerase in the following overlap extension-PCR (OE-PCR) to generate the desired sequence. Several endonucleases have been utilised for error correction (e.g. Fuhrmann *et al.*, 2005). Advantages of this strategy include (i) that it can be applied to any assembly, regardless of its sequence(s), and (ii) that it does not require unusual, dedicated hardware. However, there is no consensus regarding either the most effective method for the overall gene synthesis or the error-correction step that is performed using a separate protocol. Using current methods, a complete gene synthesis protocol consists of synthesis of the full-length gene sequence (often using two PCR steps), endonuclease mismatch cleavage and then a final OE-PCR to reassemble the gene with fewer errors. However, in developing our methods for gene synthesis, we observed that error correction becomes considerably more difficult for sequences larger than 1 kb using this method, especially during the final OE-PCR step. Moreover, there is a much more substantial issue when one wishes to synthesise protein libraries with varied, mixed bases. Mismatch-repair endonucleases cleave these variant sequences (recognised as mismatches) and this prevents their reassembly into the full-length gene. Hence, no method has been described that can incorporate variant sequences and enzymatic error correction for the synthesis of protein libraries. Therefore, the use of *de novo* gene synthesis to generate protein libraries is currently limited to only small genes.

**Fig. 1.**
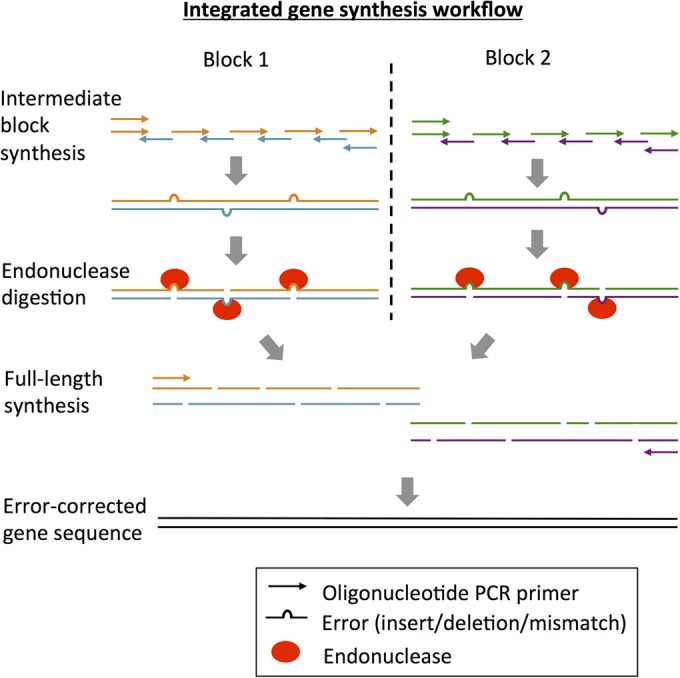
The integrated gene synthesis workflow. Synthesis of a gene using two intermediate blocks is shown as an example. Similar to previously published methods we first synthesise intermediate blocks using up to 12 oligos in one reaction. However in contrast, error correction (endonuclease cleavage) is then performed on these intermediate blocks. Pooling all these digested products together produces overlapping fragments that span the full length of the gene and therefore the gene is assembled directly in a second PCR step. This approach improves the removal of erroneous nucleotides and the assembly of full-length genes by PCR (particularly sequences greater than 1000 bp), while also reducing the number of steps compared to a typical workflow.

First, we describe an efficient protocol that integrates error correction with gene synthesis in a single workflow (Fig. 1). We show that the synthesis of enhanced green fluorescent protein (EGFP, encoded by a 747 bp gene) and a larger enzyme (monoamine oxidase-N from *Aspergillus niger*, 1518 bp) is highly efficient and therefore suitable for direct expression in *Escherichia coli* (without pre-selection) for functional screening. Following synthesis, ligation, transformation and expression directly into *E. coli*, 85% of EGFP colonies exhibited green fluorescence and 76% of MAO-N colonies showed full catalytic activity (and correct DNA sequence). Next, we introduce the SpeedyGenes method for the synthesis of diverse protein libraries with error correction. SpeedyGenes overcomes the limitation of endonuclease cleavage of the mixed bases by reintroducing the relevant oligonucleotides (encoding the variant sequences) after endonuclease treatment, thus permitting the synthesis of variant sequences while maintaining error correction. We show that this method can incorporate multiple variant oligomers concurrently to allow the screening of combinatorial variants of MAO-N. Given the accuracy of the gene synthesis, libraries can be expressed and screened without pre-selection and prior to any verification by DNA sequencing.

## Materials and methods

### Synthesis of intermediate DNA blocks from oligonucleotides

Oligonucleotides were designed using GeneGenie (Swainston *et al.*, 2014), which produces overlapping sequences with matched melting temperatures (*T*_m_). All input parameters and output sequences are shown in Supplementary Note 1. A target overlap melting temperature of 60°C and *E. coli* as the host organism were specified. For cloning after gene synthesis, 15-nt sequences were added at the 5′ and 3′ ends that correspond to target expression vector (pET16b, Novagen). Oligonucleotides were synthesised by Integrated DNA Technologies.

The first intermediate blocks were assembled using no more than 12 oligonucleotides each. For example, EGFP was encoded using 24 oligonucleotides, hence two intermediate assemblies containing 12 oligonucleotides each were synthesised. For each block, the two outermost oligonucleotides (i.e. numbers 1 and 12) were used as primers for the PCR. The remaining inner oligonucleotides (numbers 2–11) were pooled together in an equimolar mixture (600 nM each) as PCR template. The PCR contained 600 nM forward and reverse primers, 30 nM template oligonucleotide mix, 0.2 mM dNTP mix, 1× Q5 reaction buffer and 0.02 U μl^−1^ Q5 hot-start high-fidelity polymerase (New England Biolabs) in 50 μl total volume. The PCR had an initial denaturation at 98°C for 2 min, then 35 cycles of 98°C for 10 s, 60°C (or other *T*_m_) for 20 s and 72°C for 20 s. PCR products were purified using the PCR purification kit (Qiagen).

When synthesising variants using the SpeedyGenes method, the variant oligonucleotides encoding the mixed base sequences are used for the intermediate block synthesis (instead of the original wild-type sequence oligonucleotide).

### Endonuclease digestion for error correction

PCR products were diluted to 100 ng μl^−1^ concentration (assessed using a nanodrop spectrometer) in 1× HF reaction buffer (New England Biolabs). Samples were then denatured at 95°C for 2 min and slowly hybridised by stepwise reductions in temperature: 95–85°C then incubated for 1 min (2°C s^−1^ ramp rate), then lowered to 25°C at 10°C intervals (1 min incubation every 10°C, 0.3°C s^−1^ ramp rate) as previously described (Saaem *et al.*, 2012). Hybridised DNA (5 μl) was then incubated with 5 μl of Surveyor endonuclease (Transgenomic) mixture containing 2 μl Surveyor, 1 μl enhancer and 1× HF buffer and incubated for 2 h at 42°C. Samples were purified using the PCR purification kit (Qiagen) to stop the reaction.

### Synthesis of full-length genes and variant sequences

Full-length gene sequences were synthesised by pooling equal volumes of the purified block digests for use as the PCR template. The PCR had the same constituents as above, except that 2 μl of the template mix was used and the two outermost oligonucleotides (numbers 1 and 24 for EGFP) were primers. Reaction conditions were denaturation at 98°C for 2 min, followed by 35 cycles of 98°C for 10 s, 60°C (or other *T*_m_) for 20 s and 72°C for 60 s. For synthesising variants using ‘spiking’, 6 nM of the oligonucleotide containing the variant sequences is added to the PCR. Synthesised genes were purified using gel electrophoresis and the Minelute gel extraction kit (Qiagen).

### Plasmid ligation and recombinant protein expression

Synthesised genes were ligated into a linearised pET16b expression vector (Novagen) using the In-Fusion cloning kit (Clontech), following the manufacturers' protocol. Plasmids were then transformed into T7 express competent *E. coli* cells (New England Biolabs) and spread onto LB agar plates (100 μg ml^−1^ ampicillin) and incubated overnight at 37°C. For direct functional screening, cells were spread onto a Hybond-N transfer membrane, which enabled the *E. coli* transformants to be transferred to an agar plate containing 1 mM isopropyl-beta-d-thiogalactopyranoside to induce protein expression. For MAO-N activity assays, the induced bacterial colonies were analysed for oxidase activity through the production of hydrogen peroxide by the colorimetric assay outlined by Alexeeva *et al.* (2002, 2003) using α-methylbenzylamine as the substrate.

To determine the DNA sequence of the synthesised constructs, bacterial colonies were inoculated and grown in liquid culture (LB with 100 μg ml^−1^ ampicillin), followed by plasmid extraction (Spin Miniprep kit, Qiagen) and Sanger sequencing (DNA Sequencing Facility, University of Manchester).

## Results

### Development of an integrated gene synthesis workflow with error correction

All oligonucleotide sequences were designed using the online tool, GeneGenie (Swainston *et al.*, 2014). GeneGenie (http://g.gene-genie.appspot.com/) takes as its input the amino acid sequence of any protein, and incorporates user-defined parameters to facilitate DNA assembly (targeted annealing temperatures, maximum oligonucleotide lengths), downstream cloning (by adding 5′ and 3′ sequences, e.g. restriction sites) and organism-specific protein expression (via codon optimisation). All sequences used are shown in Supplementary Material; the typical oligonucleotides used here were 55–60 bases in length. Oligonucleotides designed for PCR-based assembly using other software, e.g. DNAWorks (Hoover and Lubkowski, 2002), are also fully compatible with these methods.

Gene sequences were assembled initially using PCR-based methods, following the protocol outlined by Xiong *et al.* (2004, 2008). Using this method, genes are first assembled into dsDNA blocks using a set of up to 12 overlapping oligonucleotides (here termed as intermediate assemblies). In this (earlier) protocol, genes requiring >12 oligonucleotides are assembled using a second PCR, which uses the PCR products from the first reaction as a template. To minimise any errors introduced by PCR, it is crucial to use a high-fidelity polymerase; here the Q5 polymerase (New England Biolabs) was used. As expected, the problem of sequence errors becomes more significant as gene sequences get longer, and in addition the protocol is both cumbersome and time consuming. Thus, a feature of the SpeedyGenes approach is that the error-correction procedure is performed using the shorter intermediate sequences generated directly from the oligonucleotides.

As mentioned above, the enzymatic mismatch cleavage strategy was adopted for the error-correction strategy as it provides a universal method that can be applied to any construct independent of sequence. As per the conventional protocols (e.g. Xiong *et al.*, 2004, 2008; Fuhrmann *et al.*, 2005), denaturation and slow hybridisation create mismatches where sequence errors have been generated. To remove these, samples are then incubated with a mismatch endonuclease. Three endonucleases were tested, viz. T7 endonuclease I (bacteriophage T7), endonuclease V (*E. coli*, Fuhrmann *et al.*, 2005) and Surveyor (from celery, Saaem *et al.*, 2012). Each enzyme exhibited cleavage of the synthesised blocks and was able to reduce the number of errors in the final gene sequence. However, of the three tested, Surveyor was the most effective for the removal of errors (assessed by MAO-N activity assays and following DNA sequencing) and was thus used in subsequent studies.

**Fig. 2.**
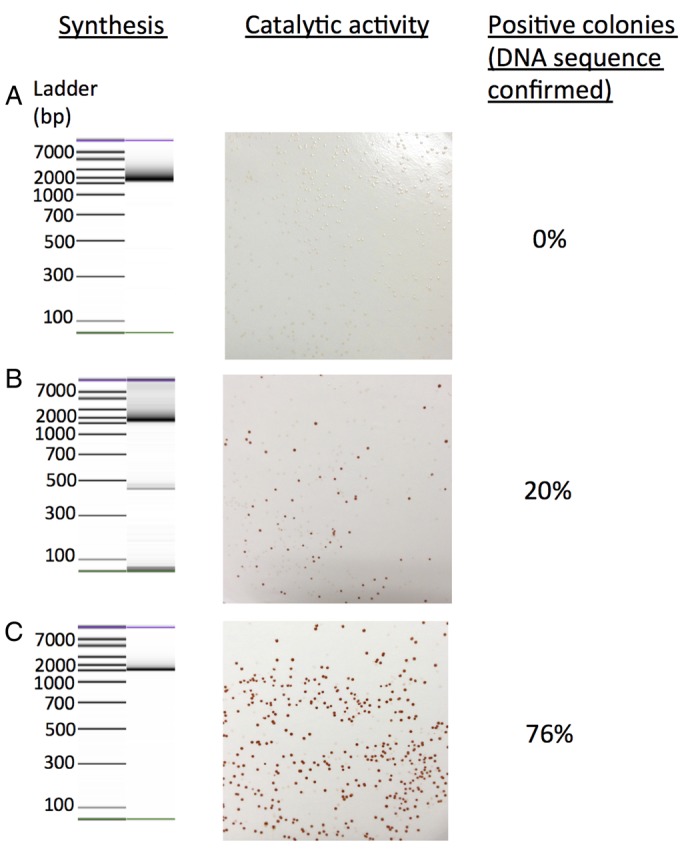
A comparison of gene synthesis error-correction techniques using microfluidic capillary electrophoresis, catalytic activity assays and DNA sequencing. (**A**) Synthesis of MAO-N with no error correction yielded highly erroneous gene sequences, illustrated by the increased amounts of longer fragments shown in the gel-like image. Expression of these sequences in *E. coli* yielded no colonies with catalytic activity and the presence of errors was identified by DNA sequencing. (**B**) Conventional Surveyor digestion of the full-length MAO-N sequence did not produce a pure product after the final OE-PCR (shown) and yielded 20% *E. coli* colonies with oxidase activity and correct sequence. (**C**) The SpeedyGenes method greatly improved the PCR synthesis of MAO-N. Expression of these sequences yielded 76% colonies with activity and correct DNA sequence.

Importantly, performing the endonuclease digestion on the intermediate blocks was found to reduce the amount of digestion, with a proportion of the sample remaining undigested (and presumably error-free). This is in contrast to digestion of the full-length sequence, where a higher amount of digestion occurs (and very few undigested products are visible) due to the incorporation of more errors as the sequence lengthens (Supplementary Note 2). As a result, this protocol can tolerate significantly longer incubation times than the conventional method (Saaem *et al.*, 2012), to enable a more thorough digestion of the samples for complete cleavage of mismatched sequences. Using Surveyor, we continued digestion for 2 h before reaction termination, in contrast to existing methods that can tolerate only a 20–60 min incubation before termination of digestion (Saaem *et al.*, 2012). Fig. 2B and C shows the difference between the two error-correction protocols. In the synthesis of the 1518 bp gene MAO-N, 76% of positive clones contained the correct sequence, whereas use of the published Surveyor method (Saaem *et al.*, 2012) yielded only 20% correct clones with the desired gene sequence (percentages defined as colonies that exhibited both full catalytic activity and correct DNA sequence from sequencing). This improvement is illustrated by the capillary electrophoresis-derived traces (Fig. 2A–C and Supplementary Note 3), which clearly show that the presented method reduces the production of erroneous sequences (i.e. those with an incorrect length).

Since less error-correcting endonuclease digestion occurs on the intermediate blocks compared to that of the full-length sequence, these block digest products contain longer fragments which overlap, and thus can be reassembled by OE-PCR more efficiently for the synthesis of the full-length gene. In particular, fragments from each digested block could be pooled and used directly for the synthesis of the full-length gene. This is in contrast to alternative methods that we considered, which reassemble the intermediate blocks after error correction but before synthesis of the gene. In the case of MAO-N (four blocks), this strategy would introduce errors from each of the eight primers used in the synthesis (two for each block). In our approach, the use of just the two outermost oligonucleotides as primers in the full-length OE-PCR synthesis minimises the introduction of new errors into the sequence. This approach also has the great benefit of reducing the number of steps required in the workflow (Fig. 1).

**Fig. 3.**
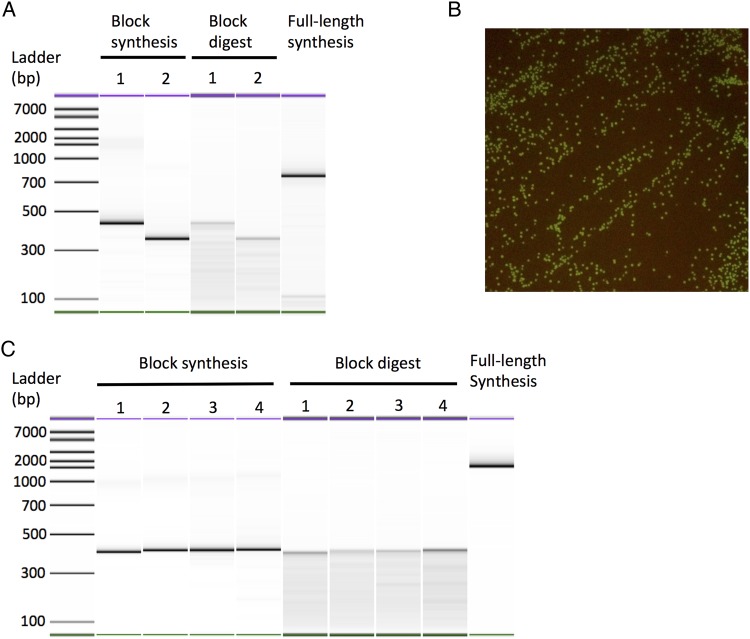
The integrated gene synthesis workflow and direct functional assay in *E. coli*. (**A**) EGFP was synthesised using two intermediate block fragments of 12 oligonucleotides which were then subjected to Surveyor digestion for 120 min. Full-length EGFP was then assembled efficiently from the digest products by OE-PCR. (**B**) Expression of the synthetic gene for EGFP in *E. coli* produced 85% colonies with green fluorescence under blue light. DNA sequencing confirmed that all fluorescent colonies contained the correct EGFP sequence. (**C**) The D5 variant of MAO-N (1518 bp) was synthesised using four intermediate fragments (labelled 1–4) using the same method. Expression of this construct is shown in Fig. 2C.

Following synthesis, the gene sequence can then be cloned or used for assembling larger DNA molecules. In the present study, genes were ligated into standard plasmids for recombinant protein expression. As a test for the efficiency of gene synthesis for screening, we selected two proteins with established functional assays that could be performed directly after synthesis (i.e. before sequence verification). For EGFP, bacterial colonies could be screened for fluorescence under UV/blue light and for MAO-N (D5 variant (Atkin *et al.*, 2008)) an *in situ* colorimetric assay using the substrate α-methylbenzylamine has been developed to screen for activity from bacterial colonies on petri plates directly (Alexeeva *et al.*, 2002; Ghislieri *et al.*, 2013; Rowles *et al.*, 2012). The expression of EGFP (747 bp, Fig. 3A and B, Supplementary Note 4) and MAO-N (1518 bp, Fig. 2C and 3C, Supplementary Note 5) yielded a high proportion of positive functional clones (85% for EGFP, 76% for MAO-N), indicating that the method is sufficiently accurate to screen for functional protein before any sequence verification is required. DNA sequencing of these positive colonies confirmed that they encoded correct, error-free sequences. This is a significant improvement over previous methods, where error correction performed on the full-length MAO-N D5 sequence yielded just 20% of colonies with full catalytic activity (Fig. 2). It is worth noting that the In-Fusion ligation system used here has a reported efficiency of 90% (Marsischky and LaBaer, 2004), so the actual accuracy of the gene synthesis method is even higher than the functional assay suggests.

### Development of the SpeedyGenes method to synthesise protein libraries

**Fig. 4.**
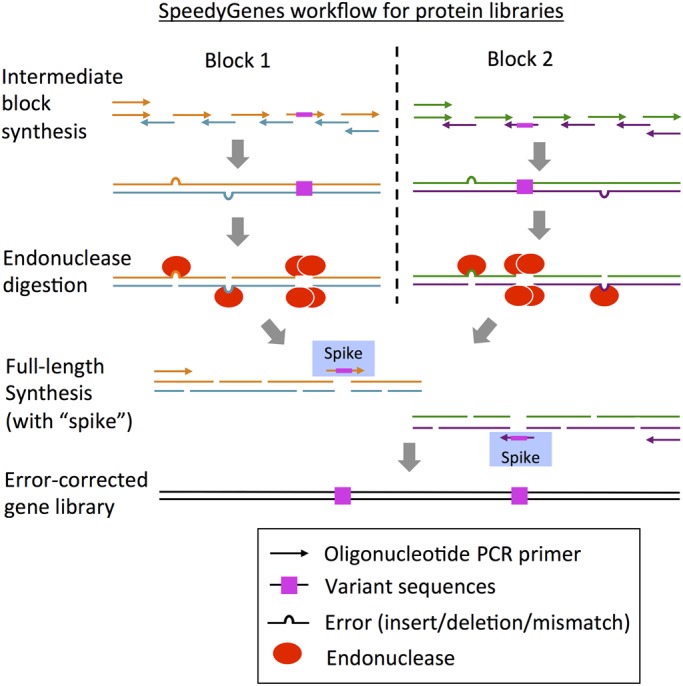
Overview of the SpeedyGenes protocol using oligonucleotide ‘spiking’ to generate protein libraries. Oligonucleotides encoding the variant (mixed base) sequences are used to synthesise the intermediate blocks in the first step of the gene synthesis protocol. However, the variant sequences are heavily digested during the endonuclease incubation, hence the oligonucleotides encoding the variant sequences are ‘spiked’ into the final PCR to synthesise the full-length gene variant.

Following establishment of the above gene synthesis workflow, our objective was to develop a method that could maintain this accuracy while also encoding variant codons (containing non-determined mixtures of bases) at different positions in the sequence. The introduction of variant bases (e.g. the code N referring to a non-discriminate mix of all four bases (Nomenclature Committee of the International Union of Biochemistry (NC-IUB), 1985), Supplementary Note 6) into a desired sequence is a fundamental technique in engineering protein sequences as it permits the generation of libraries of protein variants for the purposes of directed evolution (Reetz *et al.*, 2006*a*; Reetz and Carballeira, 2007; Steffens and Williams, 2007; Turner, 2009; Mundhada *et al.*, 2011; Steiner and Schwab, 2012). The approach described here is to use gene synthesis to create these protein libraries. Hence, the oligonucleotides encoding variant sequences (mixed bases) are used in the synthesis of the intermediate blocks (in place of the original wild-type sequence oligomers). Notably, previous error-correction methods cannot accommodate such variant sequences as the mixture of bases creates mismatches in the sequence, which are completely digested during the enzymatic error-correction step and thus prevent reassembly in the following OE-PCR. We reasoned that by reintroducing the oligonucleotide encoding the variant sequences back into the OE-PCR (‘spiking in’), this part of the sequence could be ‘bridged’ to allow the overall synthesis of gene sequences with the intended variant nucleotides. Specifically, adding 6 nM of the ‘spiking’ oligonucleotide permitted the full-length gene to be assembled successfully by OE-PCR (illustrated in Fig. 4). Using this approach, a large gene (>1 kb) could be synthesised with error correction while also introducing specific variant bases to create a protein library.

### The use of SpeedyGenes to make controlled protein mutants

**Fig. 5.**
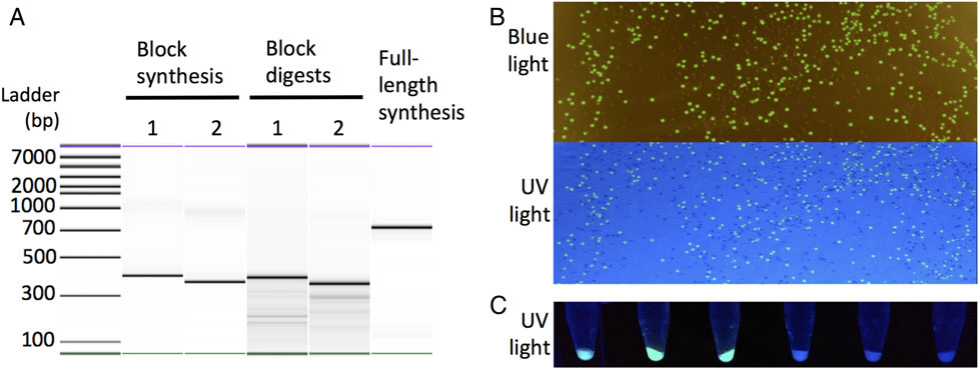
Synthesis of green and blue variants of EGFP using the SpeedyGenes method. (**A**) Oligonucleotides 6 and 12, which encoded the variant codon sequences, were ‘spiked’ into the final OE-PCR at a concentration of 6 nM each to assemble the full-length sequence. (**B**) *In situ* expression of these amplicons in *E. coli* yielded colonies with green fluorescence under blue light (EGFP, upper image) and/or blue fluorescence under UV light (EBFP, lower image). (**C**) Selected colonies from this plate were induced in liquid culture and the expression of EGFP (left-most three tubes) and EBFP (right-hand three tubes) was observed under UV light.

An important aspect of the SpeedyGenes strategy is that the mutations created are entirely encoded by the oligonucleotides used in the assembly (not random or shuffled). Hence, the user can control the specific mutations to be introduced at any point in the sequence. To illustrate this, the sequence of EGFP was synthesised containing two pre-determined variant codons. Using the knowledge that the mutations Y66H and Y145F yield the blue variant of EGFP (Rizzuto *et al.*, 1996), two codons were devised using GeneGenie at Residues 66 and 145, such that they would encode the Residues Y/H and Y/F, respectively (using the IUPAC code, for mixed sequences, see Supplementary Notes 6 and 7). Hence, in one synthesis a simple library containing both EGFP and EBFP (and the mixed) sequences would be created. These mutations were introduced into two different oligonucleotides (numbered 6 and 12) and so also demonstrate that two variant oligonucleotides can be ‘spiked in’ during a single reaction. The EGFP sequence (using 20 oligonucleotides) was assembled efficiently using two intermediate assemblies of 10 oligonucleotides, subjected to Surveyor endonuclease treatment, and oligonucleotides 6 and 12 were ‘spiked in’ (6 nM concentration) for the OE-PCR synthesis of EGFP/EBFP sequences (Fig. 5A). Amplicons were then ligated into the expression vector and expressed in colonies *in situ*. Green (Fig. 5B upper and lower) and blue (Fig. 5B dark blue, lower) colonies were clearly visible under UV light, showing that both the EGFP and EBFP had been synthesised efficiently. Selection and induction of selected colonies in liquid culture showed that individual clones exhibited green and blue fluorescence (Fig. 5C) and the correct EGFP and EBFP sequences were verified by DNA sequencing.

### The use of SpeedyGenes to synthesise protein libraries

**Fig. 6.**
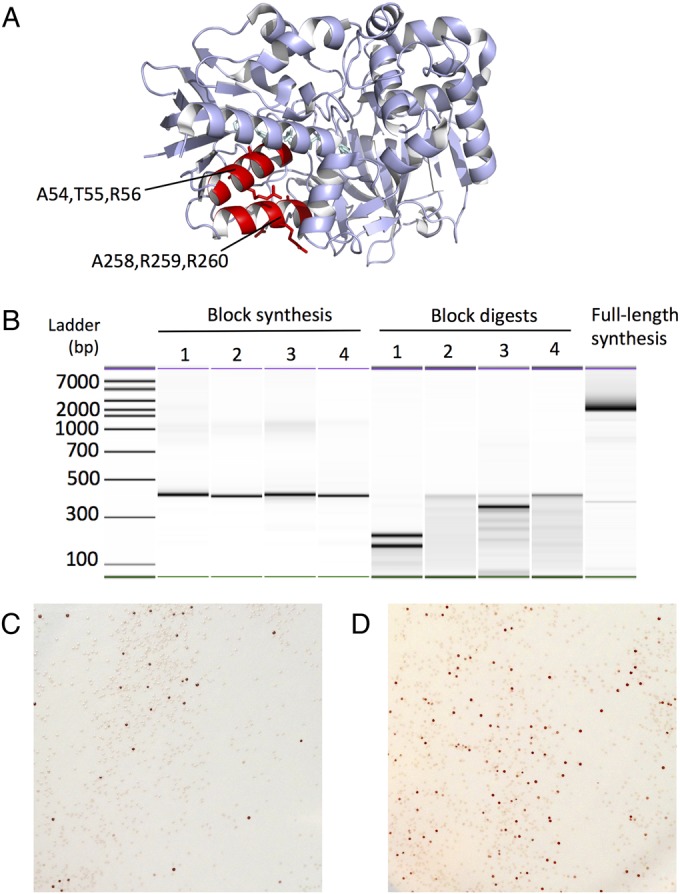
Synthesis of MAO-N variants for functional screening. (**A**) Two adjacent α-helices were selected for the generation of protein libraries (highlighted in red on the MAO-N D5 structure, PDB code: 2VVM). The ATR (Residues 54–56) and ARR (258–260) side chains are shown. (**B**) The MAO-N sequence encoding six variant codons was synthesised using the SpeedyGenes method. The observed difference in digestion of Fragments 1 and 3 is due to the cleavage of the variant sequences encoded within these fragments. Oligonucleotides 5 (encoding variant codons for residues ATR) and 22 (for ARR) were ‘spiked’ into the final OE-PCR at 6 nM prior to the full-length synthesis. (**C**) The synthesised sequence was expressed in *E. coli* and screened for MAO-N activity. (**D**) Another MAO-N variant encoding variant codons for the ATR sequence only was also synthesised and screened for MAO-N activity. Both variants show a variation in activity which were then analysed using DNA sequencing, illustrating that SpeedyGenes can synthesise variant libraries *de novo* for direct recombinant expression and functional screening.

As already described, the primary purpose of SpeedyGenes is to synthesise novel, diverse protein variants for functional screening in directed evolution studies. In its first application, variants of the MAO-N D5 gene from *Aspergillus niger* (1518 bp) were synthesised. We selected two α-helices (residues 56–8 and 258–60) that are adjacent in the protein structure (highlighted in Fig. 6A, PDB code: 2VVM), to screen combinatorial variants for residues that are interacting together. In this case 3 amino acids at each position were selected, hence 9 consecutive bases were varied using the IUPAC code. These variant sequences (encoded on oligonucleotides 5 and 22 for residues 56–8 and 258–60, respectively) were synthesised using the SpeedyGenes method and screened for monoamine oxidase activity (Fig. 6B and C, see Supplementary Note 8). This method successfully synthesised the library in a single procedure and the direct expression in *E. coli* (Fig. 6C) clearly shows that MAO-N variants of different activity were generated. DNA sequencing of the negative colonies in the assay confirmed that these encoded variants of MAO-N that had been correctly synthesised.

In this case each of the variant codons could encode 3 or 5 amino acids (at each of the two separate loci), such that a library of 1215 possible combinations was created and screened. SpeedyGenes is therefore an effective gene synthesis platform that can generate diverse, yet accurate protein libraries in a single workflow.

## Discussion

When following current published methods for gene synthesis/assembly, the steps of DNA sequence and oligonucleotide design, DNA fabrication, error correction and plasmid ligation use separately designed protocols. Here, we provide the first example of an integrated gene synthesis method that is fully complemented by an *in silico* design tool, GeneGenie (Swainston *et al.*, 2014). We describe an efficient method of gene synthesis that requires fewer steps than those described previously, thereby providing the opportunity to assemble large and accurate constructs. Moreover, these constructs may also be varied in a controlled manner for the purposes of protein engineering and directed evolution. After oligonucleotide synthesis, gene sequences containing multiple variant codons can be assembled, error-corrected and screened for functional activity *in vitro* in 3 days using the SpeedyGenes method.

The use of our gene synthesis-based approach for directed evolution offers many advantages for protein engineering. In particular, and in contrast to methods such as error-prone PCR (Zaccolo and Gherardi, 1999; Pritchard *et al.*, 2004; Drummond *et al.*, 2005; Wedge *et al.*, 2009) or DNA shuffling (Stemmer, 1994), desired mutations can be introduced at specific positions and high mutation rates can be used while at the same time preventing the introduction of unwanted mutations such as those encoding premature ‘stop’ codons. These methods are therefore complemented by random mutagenesis approaches, like error-prone PCR and DNA shuffling, where regions of interest are not identified. Thus, the use of GeneGenie to encode variant sequences coupled with their synthesis using the method described here is a powerful tool for the generation of libraries of protein variants that can then be subjected directly to functional screens for a desired application. Here we demonstrate this with the efficient synthesis and screening of a library of MAO-N mutants. Synthesis and screening of this large gene (1518 bp) without error correction would not produce libraries with any activity, while use of existing error-correction methods would not be able to assemble the gene with the desired mutations. This main objective for SpeedyGenes is thus different from that of other large DNA syntheses (i.e. genomes (Gibson *et al.*, 2009; Gibson, 2012)) that are performed without error correction and where correct clones are verified by sequencing before assembling kilobase-sized molecules.

SpeedyGenes was shown to accommodate variant sequences on two different oligonucleotides concurrently (more variant oligonucleotides were not tested here), so mutations can be introduced simultaneously to produce (and screen for) combinatorial variants. This can therefore increase dramatically the rate of knowledge-based navigation of the relevant search space (Fox and Huisman, 2008; Kell, 2012; Romero *et al.*, 2013) for functional screening, and coupled with efficient synthesis and expression in *E. coli* or any other preferred host can provide a platform for the potential screening of millions of sequence variants in a single generation.

The introduction of variant sequences using other methods such as site-directed mutagenesis (Wei *et al.*, 2004; Reetz *et al.*, 2006*b*,*c*; Turner, 2009; Reetz, 2011) is commonly used. However this is typically limited to modifying sequences in a specific position using a single oligonucleotide. In contrast, this work demonstrates an alternative strategy using *de novo* gene synthesis that can introduce multiple combinatorial variants at different positions in the sequence simultaneously and in parallel. We demonstrate this by synthesising 9 bp of variant sequences (three codons) simultaneously in two different (and in sequence space remote) positions in the MAO-N gene, followed by a direct screen for catalytic activity, creating a library of 1215 possible variants. This library was screened and the positive variants then checked by DNA sequencing. These variant sequences were easily extended up to 15 bp in our trials (longer variants have not been tested to date) and future work will include greater numbers of variant oligonucleotides in one reaction to create much larger protein variants to address a larger sequence space. A great benefit of this gene synthesis approach is that many different combinations, using a variety of different variant oligomers, can be synthesised in parallel, making this method amenable to automation and high-throughput studies. Together with GeneGenie (Swainston *et al.*, 2014), these are powerful tools for use in synthetic biology, with the ability to engineer not only any desired DNA sequence but also to introduce specific sequence variants at any position in the sequence. Therefore, these tools will greatly facilitate protein engineering for the identification of variants with novel function, altered substrate specificity or improved catalytic activity (Grünberg and Serrano, 2010; Foo *et al.*, 2012).

## Supplementary material

Supplementary material is available at *PEDS* online.

## Funding

This work was supported by the Biotechnology and Biological Sciences Research Council and the University of Manchester. Funding to pay the Open Access publication charges for this article was provided by Research Councils UK via the University of Manchester.

## Supplementary Material

Supplementary Data
