# Supplementary material for: SpeedyGenes: an improved gene synthesis method for the efficient production of error-corrected, synthetic protein libraries for directed evolution
Source: Protein Eng Des Sel. 2014 Aug 9;27(9):273–80. doi: 10.1093/protein/gzu029 (PMC4140418; doi:10.1093/protein/gzu029)
Supplement: Supplementary Data [file supp_27_9_273__index.html]

SpeedyGenes: an improved gene synthesis method for the efficient production of error-corrected, synthetic protein libraries for directed evolution — Supplementary Data 

# SpeedyGenes: an improved gene synthesis method for the efficient production of error-corrected, synthetic protein libraries for directed evolution

## Supplementary Data

Supplementary Data

**Files in this Data Supplement:**

- Supplementary Data - Docx file
