# Supplementary material for: SpeedyGenes: an improved gene synthesis method for the efficient production of error-corrected, synthetic protein libraries for directed evolution
Source: Protein Eng Des Sel. 2014 Aug 9;27(9):273–80. doi: 10.1093/protein/gzu029 (PMC4140418; doi:10.1093/protein/gzu029)
Supplement: Supplementary Data [file supp_gzu029_gzu029supp.docx]

**Supplementary Material**

**Supplementary Note 1. GeneGenie input parameters used for all constructs:**

5’ cloning sequence: TCGAAGGTCGTCATATG

3’ cloning sequence: TAAGGATCCGGCTGCTAAC

Maximum oligonucleotide length: 60

Melting temperature (Tm) / °C: 60

Host organism: Escherichia coli

**Supplementary Note 2. Comparison of Surveyor endonuclease digestion of the large MAO-N gene on the full-length and intermediate blocks**

**
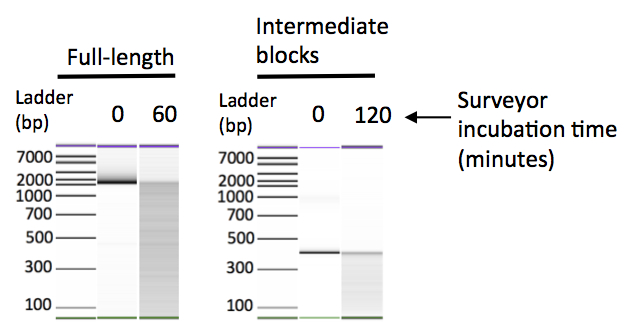
**

**Supplementary Note 3. Capillary electrophoresis electropherogram traces of MAO-N error correction**

**
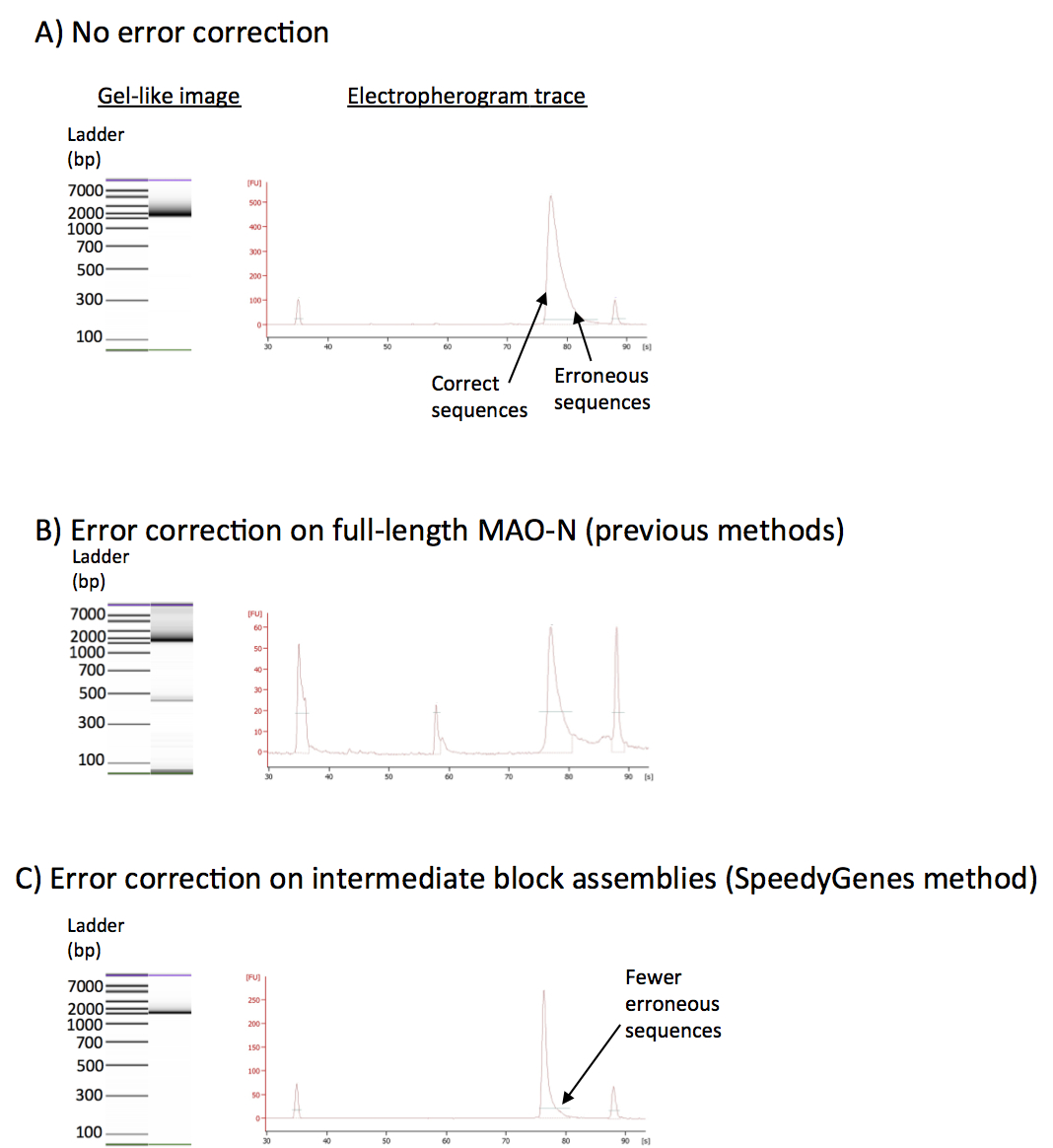
**

**Supplementary Note 4. Synthesis of enhanced green fluorescent protein**

Inputted protein sequence:

VSKGEELFTGVVPILVELDGDVNGHKFSVSGEGEGDATYGKLTLKFICTTGKLPVPWPTLVTTLTYGVQCFSRYPDHMKQHDFFKSAMPEGYVQERTIFFKDDGNYKTRAEVKFEGDTLVNRIELKGIDFKEDGNILGHKLEYNYNSHNVYIMADKQKNGIKVNFKIRHNIEDGSVQLADHYQQNTPIGDGPVLLPDNHYLSTQSALSKDPNEKRDHMVLLEFVTAAGITLGMDELYK

DNA sequence output:

TCGAAGGTCG TCATATGGTT AGTAAAGGTG AAGAACTGTT TACCGGTGTT

GTTCCGATTC TGGTGGAGCT GGATGGTGAT GTTAATGGTC ATAAATTTAG

CGTTAGCGGT GAAGGCGAAG GTGATGCAAC CTATGGTAAA CTGACACTGA

AATTTATTTG TACCACCGGT AAACTGCCGG TTCCGTGGCC GACCCTGGTG

ACAACCCTGA CCTATGGTGT TCAGTGTTTT AGCCGTTATC CGGATCACAT

GAAACAGCAT GATTTTTTTA AATCTGCAAT GCCCGAAGGT TATGTTCAGG

AACGCACCAT TTTTTTTAAA GATGATGGCA ATTATAAAAC CCGTGCAGAA

GTTAAATTTG AAGGTGATAC CCTGGTTAAT CGTATTGAAC TGAAAGGTAT

CGATTTTAAA GAAGATGGTA ATATTCTGGG CCATAAACTG GAATATAATT

ATAATTCTCA TAATGTGTAT ATTATGGCTG ATAAACAGAA AAATGGCATT

AAAGTTAATT TTAAAATTCG GCACAATATT GAAGATGGTA GTGTTCAGCT

GGCAGATCAT TATCAGCAGA ATACTCCGAT TGGTGATGGT CCGGTGCTGC

TGCCTGATAA TCATTATCTG AGCACCCAGA GCGCGCTGAG CAAAGATCCG

AATGAAAAAC GCGATCATAT GGTTCTGCTG GAATTTGTTA CGGCAGCAGG

TATTACGCTG GGTATGGATG AACTGTACAA ATAAGGATCC GGCTGCTAAC

Oligonucleotide sequences:

| Oligonucleotide | Length |
| --- | --- |
| 1 | TCGAAGGTCGTCATATGGTTAGTAAAGGTGAAGAACTGTTTACCGGTGTTGTTCCGAT |
| 2 | TGACCATTAACATCACCATCCAGCTCCACCAGAATCGGAACAACACCGGTAA |
| 3 | TGGATGGTGATGTTAATGGTCATAAATTTAGCGTTAGCGGTGAAGGCGAAGGTGATG |
| 4 | GGTACAAATAAATTTCAGTGTCAGTTTACCATAGGTTGCATCACCTTCGCCTTCAC |
| 5 | TAAACTGACACTGAAATTTATTTGTACCACCGGTAAACTGCCGGTTCCGTGGCCGACC |
| 6 | CGGCTAAAACACTGAACACCATAGGTCAGGGTTGTCACCAGGGTCGGCCACGGAAC |
| 7 | ATGGTGTTCAGTGTTTTAGCCGTTATCCGGATCACATGAAACAGCATGATTTTTTTAA |
| 8 | CTGAACATAACCTTCGGGCATTGCAGATTTAAAAAAATCATGCTGTTTCATGTGAT |
| 9 | GCCCGAAGGTTATGTTCAGGAACGCACCATTTTTTTTAAAGATGATGGCAATTATA |
| 10 | TCAAATTTAACTTCTGCACGGGTTTTATAATTGCCATCATCTTTAAAAAAAATGGT |
| 11 | CCCGTGCAGAAGTTAAATTTGAAGGTGATACCCTGGTTAATCGTATTGAACTGAAAGG |
| 12 | CAGAATATTACCATCTTCTTTAAAATCGATACCTTTCAGTTCAATACGATTAACCA |
| 13 | TCGATTTTAAAGAAGATGGTAATATTCTGGGCCATAAACTGGAATATAATTATAATTCTC |
| 14 | GTTTATCAGCCATAATATACACATTATGAGAATTATAATTATATTCCAGTTTATGGC |
| 15 | CATAATGTGTATATTATGGCTGATAAACAGAAAAATGGCATTAAAGTTAATTTTAA |
| 16 | ATCTTCAATATTGTGCCGAATTTTAAAATTAACTTTAATGCCATTTTTCTGTTTAT |
| 17 | TAATTTTAAAATTCGGCACAATATTGAAGATGGTAGTGTTCAGCTGGCAGATCATT |
| 18 | GCACCGGACCATCACCAATCGGAGTATTCTGCTGATAATGATCTGCCAGCTGAAC |
| 19 | GGTGATGGTCCGGTGCTGCTGCCTGATAATCATTATCTGAGCACCCAGAGCGCG |
| 20 | GAACCATATGATCGCGTTTTTCATTCGGATCTTTGCTCAGCGCGCTCTGGGTGCT |
| 21 | TGAAAAACGCGATCATATGGTTCTGCTGGAATTTGTTACGGCAGCAGGTATTACGCTG |
| 22 | GCAGCCGGATCCTTATTTGTACAGTTCATCCATACCCAGCGTAATACCTGCTGC |
| 23 | TACAAATAAGGATCCGGCTGCTAAC |
| 24 | GTTAGCAGCCGGATCC |

**Supplementary Note 5. Monoamine oxidase-N (D5 variant)**

Protein sequence:

TSRDGYQWTPETGLTQGVPSLGVISPPTNIEDTDKDGPWDVIVIGGGYCGLTATRDLTVAGFKTLLLEARDRIGGRSWSSNIDGYPYEMGGTWVHWHQSHVWREITRYKMHNALSPSFNFSRGVNHFQLRTNPTTSTYMTHEAEDELLRSALHKFTNVDGTNGRTVLPFPHDMFYVPEFRKYDEMSYSERIDQIRDELSLNERSSLEAFILLCSGGTLENSSFGEFLHWWAMSGYTYQGCMDCLMSYKFKDGQSAFARRFWEEAAGTGRLGYVFGCPVRSVVNERDAARVTARDGREFVAKRVVCTIPLNVLSTIQFSPALSTERISAMQAGHVSMCTKVHAEVDNKDMRSWTGIAYPFNKLCYAIGDGTTPAGNTHLVCFGNSANHIQPDEDVRETLKAVGQLAPGTFGVKRLVFHNWVKDEFAKGAWFFSRPGMVSECLQGLREKHGGVVFANSDWALGWRSFIDGAIEEGTRAARVVLEELGTKREVKARL

DNA sequence output:

TCGAAGGTCG TCATATGACC AGTCGTGATG GCTATCAATG GACTCCGGAA

ACCGGTCTGA CCCAGGGTGT TCCGTCTCTG GGTGTAATTA GCCCGCCGAC

TAATATTGAA GATACGGATA AAGATGGTCC GTGGGACGTG ATTGTTATTG

GCGGTGGCTA TTGTGGTCTC ACAGCAACTC GTGATCTGAC CGTGGCAGGT

TTTAAAACAC TGCTGCTGGA AGCACGCGAT CGTATTGGTG GACGCTCCTG

GTCTAGTAAT ATTGATGGTT ATCCGTATGA AATGGGCGGT ACCTGGGTTC

ATTGGCATCA GAGTCATGTC TGGCGTGAAA TTACCCGTTA TAAAATGCAT

AATGCCCTGA GTCCGAGCTT TAATTTTAGC CGTGGTGTTA ATCATTTTCA

GCTGCGTACC AATCCGACCA CATCTACATA TATGACTCAT GAAGCAGAAG

ATGAACTGCT GCGTAGTGCC CTGCATAAAT TTACCAATGT TGATGGTACT

AACGGCCGTA CCGTTTTGCC ATTTCCGCAT GATATGTTTT ATGTTCCGGA

ATTTCGTAAA TATGATGAAA TGAGTTATAG TGAACGCATT GATCAGATCC

GTGATGAACT GTCTCTGAAT GAAAGATCTA GTTTAGAAGC ATTTATCCTT

CTGTGTAGTG GTGGCACTCT GGAAAATAGC AGCTTTGGTG AATTTCTGCA

TTGGTGGGCC ATGAGCGGCT ATACGTATCA GGGTTGTATG GATTGTTTGA

TGTCATATAA ATTTAAAGAT GGCCAGAGCG CGTTCGCGCG TCGCTTTTGG

GAAGAAGCGG CAGGTACCGG TCGCCTGGGT TATGTCTTTG GCTGTCCTGT

TCGTAGTGTC GTGAACGAAC GTGATGCCGC CCGCGTTACG GCTCGCGATG

GCCGTGAATT TGTGGCGAAA CGTGTTGTGT GTACAATTCC ACTGAACGTT

CTGAGCACAA TTCAGTTTTC TCCAGCATTA AGCACGGAAC GCATTTCAGC

CATGCAGGCC GGTCATGTTA GCATGTGTAC CAAAGTGCAT GCAGAAGTGG

ACAATAAAGA TATGCGTAGT TGGACCGGTA TTGCATATCC GTTTAATAAA

CTGTGCTATG CAATTGGTGA TGGTACCACA CCAGCGGGTA ATACCCATCT

GGTGTGTTTT GGCAATAGTG CAAATCATAT ACAGCCTGAT GAAGATGTGA

GAGAAACACT GAAAGCCGTC GGTCAGTTAG CACCAGGTAC CTTTGGTGTT

AAACGTCTGG TTTTTCATAA TTGGGTTAAA GATGAATTCG CCAAAGGTGC

ATGGTTTTTC TCACGCCCGG GCATGGTGTC AGAATGTCTG CAAGGTCTGC

GGGAAAAACA TGGTGGTGTT GTTTTTGCTA ATTCTGATTG GGCCCTGGGT

TGGCGTAGTT TTATTGATGG TGCGATTGAA GAAGGTACAC GTGCAGCTCG

TGTTGTTCTG GAAGAATTAG GTACAAAACG TGAGGTTAAA GCACGCCTGT

AAGGATCCGG CTGCTAAC

Oligonucleotide sequences:

| Oligonucleotide | Sequence |
| --- | --- |
| 1 | TCGAAGGTCGTCATATGACCAGTCGTGATGGCTATCAATGGACTCCGGAAACCGGT |
| 2 | GGGCTAATTACACCCAGAGACGGAACACCCTGGGTCAGACCGGTTTCCGGAGT |
| 3 | TCTGGGTGTAATTAGCCCGCCGACTAATATTGAAGATACGGATAAAGATGGTC |
| 4 | ACAATAGCCACCGCCAATAACAATCACGTCCCACGGACCATCTTTATCCGTATCTTC |
| 5 | TGGCGGTGGCTATTGTGGTCTCACAGCAACTCGTGATCTGACCGTGGCAGGTTTTAAA |
| 6 | GTCCACCAATACGATCGCGTGCTTCCAGCAGCAGTGTTTTAAAACCTGCCACGG |
| 7 | CGATCGTATTGGTGGACGCTCCTGGTCTAGTAATATTGATGGTTATCCGTATGAAATG |
| 8 | ATGACTCTGATGCCAATGAACCCAGGTACCGCCCATTTCATACGGATAACCATCAATAT |
| 9 | TCATTGGCATCAGAGTCATGTCTGGCGTGAAATTACCCGTTATAAAATGCATAATGCCCT |
| 10 | ATTAACACCACGGCTAAAATTAAAGCTCGGACTCAGGGCATTATGCATTTTATAA |
| 11 | TTTAATTTTAGCCGTGGTGTTAATCATTTTCAGCTGCGTACCAATCCGACCACATCTA |
| 12 | TACGCAGCAGTTCATCTTCTGCTTCATGAGTCATATATGTAGATGTGGTCGGATTG |
| 13 | AAGATGAACTGCTGCGTAGTGCCCTGCATAAATTTACCAATGTTGATGGTACTAAC |
| 14 | AAAACATATCATGCGGAAATGGCAAAACGGTACGGCCGTTAGTACCATCAACATTGGTAA |
| 15 | CCATTTCCGCATGATATGTTTTATGTTCCGGAATTTCGTAAATATGATGAAATGAGTTAT |
| 16 | CAGTTCATCACGGATCTGATCAATGCGTTCACTATAACTCATTTCATCATATTTACGA |
| 17 | TCAGATCCGTGATGAACTGTCTCTGAATGAAAGATCTAGTTTAGAAGCATTTATC |
| 18 | TGCTATTTTCCAGAGTGCCACCACTACACAGAAGGATAAATGCTTCTAAACTAGATCTTT |
| 19 | GCACTCTGGAAAATAGCAGCTTTGGTGAATTTCTGCATTGGTGGGCCATGAGCGGCT |
| 20 | TAAATTTATATGACATCAAACAATCCATACAACCCTGATACGTATAGCCGCTCATGGC |
| 21 | TATGGATTGTTTGATGTCATATAAATTTAAAGATGGCCAGAGCGCG |
| 22 | CCAGGCGACCGGTACCTGCCGCTTCTTCCCAAAAGCGACGCGCGAACGCGCTCTGGCCA |
| 23 | ACCGGTCGCCTGGGTTATGTCTTTGGCTGTCCTGTTCGTAGTGTCGTGAACG |
| 24 | CACGGCCATCGCGAGCCGTAACGCGGGCGGCATCACGTTCGTTCACGACACTACGA |
| 25 | CGCGATGGCCGTGAATTTGTGGCGAAACGTGTTGTGTGTACAATTCCACTGAACGT |
| 26 | GTGCTTAATGCTGGAGAAAACTGAATTGTGCTCAGAACGTTCAGTGGAATTGTACA |
| 27 | AGTTTTCTCCAGCATTAAGCACGGAACGCATTTCAGCCATGCAGGCCGGTCATGTTA |
| 28 | CTTTATTGTCCACTTCTGCATGCACTTTGGTACACATGCTAACATGACCGGCCTG |
| 29 | CATGCAGAAGTGGACAATAAAGATATGCGTAGTTGGACCGGTATTGCATATCCGTTTAAT |
| 30 | TGTGGTACCATCACCAATTGCATAGCACAGTTTATTAAACGGATATGCAATACC |
| 31 | AATTGGTGATGGTACCACACCAGCGGGTAATACCCATCTGGTGTGTTTTGGCAATAGT |
| 32 | GTGTTTCTCTCACATCTTCATCAGGCTGTATATGATTTGCACTATTGCCAAAACACACCA |
| 33 | ATGAAGATGTGAGAGAAACACTGAAAGCCGTCGGTCAGTTAGCACCAGGTACCTTTG |
| 34 | CATCTTTAACCCAATTATGAAAAACCAGACGTTTAACACCAAAGGTACCTGGTGCTAA |
| 35 | GTTTTTCATAATTGGGTTAAAGATGAATTCGCCAAAGGTGCATGGTTTTTCTCACGCCC |
| 36 | GTTTTTCCCGCAGACCTTGCAGACATTCTGACACCATGCCCGGGCGTGAGAAAAACCAT |
| 37 | AAGGTCTGCGGGAAAAACATGGTGGTGTTGTTTTTGCTAATTCTGATTGGGCCCTG |
| 38 | CTTCTTCAATCGCACCATCAATAAAACTACGCCAACCCAGGGCCCAATCAGAATT |
| 39 | GATGGTGCGATTGAAGAAGGTACACGTGCAGCTCGTGTTGTTCTGGAAGAATTAGGTA |
| 40 | GGATCCTTACAGGCGTGCTTTAACCTCACGTTTTGTACCTAATTCTTCCAGAACAA |
| 41 | ACGCCTGTAAGGATCCGGCTGCTAAC |
| 42 | GTTAGCAGCCGGATCC |

**Supplementary Note 6. IUPAC codes**

| **Symbol** | **Nucleotide base** |
| --- | --- |
| G | G |
| A | A |
| T | T |
| C | C |
| R | G, A |
| Y | T, C |
| M | A, C |
| K | G, T |
| S | G, C |
| W | A, T |
| H | A, C, T |
| B | G, T, C |
| V | G, C, A |
| D | G, A, T |
| N | G, A, T, C |

**Supplementary Note 7. EGFP green/blue variant**

Protein sequence:

VSKGEELFTGVVPILVELDGDVNGHKFSVSGEGEGDATYGKLTLKFICTTGKLPVPWPTLVTTLTYGVQCFSRYPDHMKQHDFFKSAMPEGYVQERTIFFKDDGNYKTRAEVKFEGDTLVNRIELKGIDFKEDGNILGHKLEYNYNSHNVYIMADKQKNGIKVNFKIRHNIEDGSVQLADHYQQNTPIGDGPVLLPDNHYLSTQSALSKDPNEKRDHMVLLEFVTAAGITLGMDELYK

Specified variant sequences:

Y66 – specified variant codon YAT

Y145 – specified variant codon TWT

DNA sequence output:

TCGAAGGTCG TCATATGGTT AGCAAAGGTG AAGAACTGTT TACGGGTGTG

GTGCCGATTC TGGTTGAACT GGATGGTGAC GTTAATGGCC ACAAATTTTC

TGTTAGCGGT GAAGGTGAGG GCGATGCCAC CTATGGTAAA TGACCCTGA

AATTTATTTG CACAACCGGC AAACTGCCGG TTCCGTGGCC TACACTGGTT

ACCACCCTGA CAYATGGTGT TCAGTGCTTT AGCCGTTATC CGGATCATAT

GAAACAGCAT GATTTCTTCA AATCTGCAAT GCCGGAAGGT TATGTTCAGG

AACGTACCAT TTTCTTCAAA GACGATGGTA ATTATAAGAC CCGCGCAGAA

GTTAAATTTG AAGGTGATAC CCTGGTGAAT CGTATTGAGC TGAAAGGTAT

TGATTTTAAA GAAGATGGCA ATATTTTGGG TCATAAACTG GAATATAATT

WTAACAGCCA CAATGTGTAC ATTATGGCAG ATAAACAAAA GAACGGTATT

AAAGTGAATT TTAAAATTCG TCACAACATT GAGGATGGCA GCGTTCAGCT

GGCGGACCAT TATCAACAGA ATACCCCGAT TGGTGATGGC CGGTCTTAC

TGCCTGATAA CCATTATCTG AGCACCCAGA GCGCACTGAG CAAGGATCCT

AATGAGAAAC GTGATCATAT GGTGCTGCTG GAATTTGTGA CGCAGCAGG

TATTACCCTG GGTATGGACG AACTGTACAA ATAAGGATCC GGCTGCTAAC

Oligonucleotide sequences:

| Oligonucleotide | Sequence |
| --- | --- |
| 1 | TCGAAGGTCGTCATATGGTTAGCAAAGGTGAAGAACTGTTTACGGGTGTGGTGCC |
| 2 | AAAATTTGTGGCCATTAACGTCACCATCCAGTTCAACCAGAATCGGCACCACACCCG |
| 3 | ACGTTAATGGCCACAAATTTTCTGTTAGCGGTGAAGGTGAGGGCGATGCCACC |
| 4 | TTGCCGGTTGTGCAAATAAATTTCAGGGTCAGTTTACCATAGGTGGCATCGCCCT |
| 5 | ATTTGCACAACCGGCAAACTGCCGGTTCCGTGGCCTACACTGGTTACCAC |
| 6 | CGGATAACGGCTAAAGCACTGAACACCATRTGTCAGGGTGGTAACCAGTGTAGG |
| 7 | GCTTTAGCCGTTATCCGGATCATATGAAACAGCATGATTTCTTCAAATCTGCAATGCCG |
| 8 | CGTCTTTGAAGAAAATGGTACGTTCCTGAACATAACCTTCCGGCATTGCAGATTTGAAGA |
| 9 | GTACCATTTTCTTCAAAGACGATGGTAATTATAAGACCCGCGCAGAAGTTAAAT |
| 10 | ACCTTTCAGCTCAATACGATTCACCAGGGTATCACCTTCAAATTTAACTTCTGCGCGG |
| 11 | CGTATTGAGCTGAAAGGTATTGATTTTAAAGAAGATGGCAATATTTTGGGTCATAAA |
| 12 | ATGTACACATTGTGGCTGTTAWAATTATATTCCAGTTTATGACCCAAAATATTGCC |
| 13 | CAGCCACAATGTGTACATTATGGCAGATAAACAAAAGAACGGTATTAAAGTGAATTTT |
| 14 | CTGCCATCCTCAATGTTGTGACGAATTTTAAAATTCACTTTAATACCGTTCTTTT |
| 15 | CAACATTGAGGATGGCAGCGTTCAGCTGGCGGACCATTATCAACAGA |
| 16 | GGTTATCAGGCAGTAAGACCGGGCCATCACCAATCGGGGTATTCTGTTGATAATGGTCCG |
| 17 | GTCTTACTGCCTGATAACCATTATCTGAGCACCCAGAGCGCACTGAGCAAGG |
| 18 | TCACAAATTCCAGCAGCACCATATGATCACGTTTCTCATTAGGATCCTTGCTCAGTGCGC |
| 19 | TGCTGCTGGAATTTGTGACCGCAGCAGGTATTACCCTGGGTATGGACGAACTGTACAA |
| 20 | GTTAGCAGCCGGATCCTTATTTGTACAGTTCGTCCATAC |

**Supplementary Note 8. MAO-N D5 ATR+ARR variant**

Oligonucleotides for MAO-N D5 were the same as outlined in Supplementary Note 3.

Variant oligonucleotides were:

Oligonucleotide 5 for variant ATR:

TGGCGGTGGCTATTGTGGTCTCACAGBNWVYMRNGATCTGACCGTGGCAGGTTTTAAA

Containing the variant code for residues A (3 possible residues), T (5 possible) and R (3 possible).

Oligonucleotide 22 for variant ARR:

CCAGGCGACCGGTACCTGCCGCTTCTTCCCAAAANYKNYKNVCGAACGCGCTCTGGCCA

Containing the variant code for residues A (3 possible residues), R (3 possible) and R (3 possible).
